# Association between non-high-density lipoprotein cholesterol to high-density lipoprotein cholesterol ratio and the risk of early-onset post-stroke depression: a prospective study

**DOI:** 10.3389/fneur.2025.1645765

**Published:** 2025-09-15

**Authors:** Mingzhu Deng, Kangping Song, Lichen Gao, Zhen Wang, Wei Zhao, Wei Xu, Tieqiao Feng, Fangyi Li

**Affiliations:** ^1^Department of Neurology, The Second People’s Hospital of Hunan Province (Brain Hospital of Hunan Province), Changsha, Hunan, China; ^2^Department of Neurology, The Affiliated Changsha Central Hospital, Hengyang Medical School, University of South China, Changsha, Hunan, China; ^3^Department of Pharmacy, School of Pharmacy, Phase I Clinical Trial Centre, The Affiliated Changsha Central Hospital, Hengyang Medical School, University of South China, Changsha, China

**Keywords:** post-stroke depression, acute ischemic stroke, non-high-density lipoprotein cholesterol to high-density lipoprotein cholesterol ratio, abnormal lipid metabolism, depression

## Abstract

**Background:**

The non-high-density lipoprotein cholesterol to high-density lipoprotein cholesterol ratio (NHHR) is a recently emerging composite biomarker of atherogenic lipid metabolism. However, the relationship between NHHR and early-onset post-stroke depression (PSD) remains underexplored.

**Methods:**

Early-onset PSD was diagnosed 2 weeks after an acute ischemic stroke (AIS). Depression severity was assessed using the Hamilton Depression Scale-17 items (HAMD-17). Patients with scores ≥7 were classified into the early-onset PSD group. Spearman rank correlation analysis was used to examine the relationship between NHHR and HAMD scores. Logistic regression analysis was conducted to evaluate the association between NHHR and early-onset PSD. Sensitivity analyses were performed to assess the robustness of the results. The predictive performance of NHHR for early-onset PSD was evaluated using receiver operating characteristic curve (ROC) analysis.

**Results:**

Among 846 prospectively enrolled patients, 283 (33.45%) were diagnosed with early-onset PSD. NHHR was positively correlated with HAMD-17 scores (*r* = 0.498, *p* < 0.001). Binary logistic regression indicated that NHHR (odds ratio [OR], 1.796; 95% confidence interval [CI] 1.452–1.996, *p* < 0.001) was an independent risk factor for early-onset PSD. The area under the curve (AUC) for NHHR in predicting early-onset PSD was 0.798.

**Conclusion:**

The findings suggest that NHHR may serve as an independent risk factor for early-onset PSD, providing valuable insights for preventive strategies and prognostic management in these patients.

## Introduction

Stroke continues to be the second greatest cause of mortality globally and the main cause of long-term impairment in people (1). Many stroke survivors continue to experience long-term disability although the fatality rate from ischemic stroke has been considerably decreased by the use of intravenous thrombolysis and endovascular therapy (2). With around 35% of stroke patients developing depression at some point in their lifetime, depression is the most common neuropsychiatric side effect connected with stroke (3, 4). Post-stroke depression (PSD) has been shown to have a negative impact on survivors’ quality of life and long-term rehabilitation. Patients with PSD also have higher death rates and are more likely to experience cognitive and functional deficits (5–7). Early-onset PSD denotes patients displaying depressive symptoms within 2 weeks following the onset of an acute stroke (8–10). Early-onset PSD exhibits a greater prevalence of depressive symptoms and is significantly associated with a heightened risk of adverse outcomes in comparison to late-onset PSD (11). Consequently, identifying new biomarkers for assessing the risk of early-onset PSD is essential for improving prevention and treatment strategies.

Lipid-related ratios are not only markers of vascular risk (12–14) but also predictors of adverse cognitive and functional recovery. A recent study showed that the potential for the early predictive value of medical conditions with chronic lipid dyshomeostasis for the risk of depression and cognitive decline (15). A growing body of research links disruptions in lipid metabolism to the development of PSD. According to a prospective study, there is a correlation between elevated PSD risk and lower levels of high-density lipoprotein cholesterol (HDL-C) and higher ratios of low-density lipoprotein cholesterol (LDL-C) to HDL-C levels (16). Another study identified apolipoprotein A1, HDL-C, intestinal fatty acid binding protein, and lipoprotein (a) as potential biomarkers for PSD (6). Additionally, the ratio of monocytes to HDL-C is known to be a new composite inflammatory measure; higher levels are linked to an increased risk of developing PSD (17). The non-high-density lipoprotein cholesterol to high-density lipoprotein cholesterol ratio (NHHR) serves as a novel composite indicator for assessing atherogenic lipids (18). The NHHR offers a more effective evaluation of cardiovascular and cerebrovascular disease risk compared to other lipid parameters (19). According to earlier studies, among American adults, NHHR is a separate risk factor for depression and suicidal thoughts (20–22). Infertility and depression among American women were partially mediated by NHHR, which was also strongly correlated with the severity of depression (23). Body mass index (BMI) and depression risk are correlated, and the relationship between BMI and depression is significantly mediated by NHHR (24). Moreover, a recent cross-sectional investigation revealed a substantial correlation between NHHR and an elevated risk of PSD in adult Americans (25). Further large-scale prospective studies are needed to confirm the causal relationship between NHHR and PSD. Moreover, the association between the NHHR and early-onset PSD remains inadequately studied and lacks substantial evidence.

Early-onset PSD is marked by a higher prevalence of depressive symptoms and is significantly linked to an increased likelihood of adverse outcomes (26). The relationship between NHHR and early-onset PSD is not yet well-defined due to a lack of conclusive evidence. This prospective study investigates the association between NHHR and early-onset PSD.

## Materials and methods

### Study design and participants

**Figure 1:**
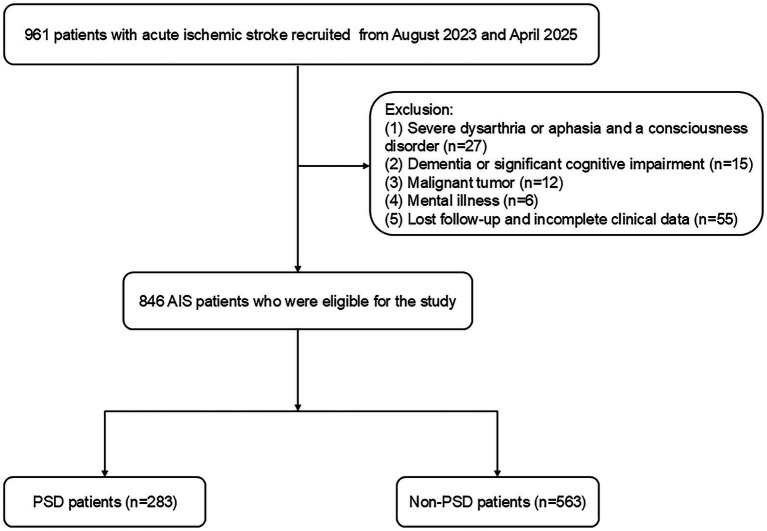
Flowchart of the study participant selection process. PSD, post-stroke depression; AIS, acute ischemic stroke.

This study was approved by Changsha Central Hospital’s Ethics Committee. From August 2023 to April 2025, Changsha Central Hospital prospectively included patients with acute ischemic stroke (AIS). The following patients met the inclusion criteria: (1) those who were between the ages of 18 and 85; (2) those who met the diagnostic criteria for ischemic stroke as stated in the Chinese guidelines for diagnosis and treatment of acute ischemic stroke 2018 (27); and (3) those who were admitted to the hospital within 72 h of the stroke onset. Exclusion criteria: (1) patients with dysarthria or aphasia and a consciousness disorder that made it difficult for them to complete tests and questionnaires; (2) patients with dementia or significant cognitive impairment before the stroke; (3) patients with severe heart, liver, or renal insufficiency; (4) patients with a mental illness, such as depression, or who were using psychotropic drugs before the stroke; (5) patients with a history of other diseases in the central nervous system, like Parkinson’s disease or epilepsy; (6) patients with malignant tumors; and (7) patients with lost follow-up and incomplete clinical data. Between August 2023 and April 2025, we enrolled 846 AIS patients (Figure 1).

### Data collection

Upon admission, all participants underwent standardized assessment of demographic characteristics (age, sex), body mass index (BMI), and vascular risk factors—including hypertension, coronary artery disease, atrial fibrillation, diabetes mellitus, as well as current smoking and drinking status—along with routine laboratory testing. Current smoking was defined as consumption of at least 10 cigarettes per day for the preceding 5 years. Current drinking was defined as consistent alcohol consumption over 5 years with an intake of at least 20 grams of ethanol per day. Stroke severity was evaluated by experienced neurologists using the National Institutes of Health Stroke Scale (NIHSS), with scores recorded within 24 h of admission. Functional status was assessed at discharge using the Barthel Index (BI), and again at one-month follow-up using the modified Rankin Scale (mRS). Imaging and diagnostic studies—including computed tomography, magnetic resonance imaging, echocardiography, electrocardiography, carotid ultrasonography, and transcranial Doppler—were performed to determine lesion location and classify stroke subtype.

Blood samples were collected from all patients within 72 h after stroke onset. Following an overnight fast of at least 8 h, venous blood was drawn between 6:00 and 7:00 a.m. the next morning. Complete blood count, including white blood cell (WBC) count, was performed using an automated hematology analyzer (BZ6800, China). Standard biochemical parameters-including creatinine (Cr), uric acid (UA), triglycerides (TG), total cholesterol (TC), high-density lipoprotein cholesterol (HDL-C), and low-density lipoprotein cholesterol (LDL-C)-were measured using an automated biochemical analyzer (HITACHI 7600, Japan). Each blood sample was analyzed in triplicate.

### Definition of early-onset PSD and NHHR measurement

Two weeks after AIS, patients were assessed for early-onset PSD by certified neurologists and psychiatrists according to the Diagnostic and Statistical Manual of Mental Disorders, 5th Edition (DSM-5). The severity of depressive symptoms was evaluated using the Hamilton Depression Scale 17 items (HAMD-17). Participants with HAMD-17 scores < 7 were classified into the non-PSD group, while those with scores ≥ 7 were assigned to the early-onset PSD group. Mild depression is defined by a score of 7–17, moderate depression by 18–23, and severe depression by greater than 24 (28). The non-HDL-C level was calculated by subtracting the HDL-C level from the TC level. The NHHR was then computed using the formula: NHHR = non-HDL-C/HDL-C (20).

### Statistical analysis

The normality of all variables was assessed using the Kolmogorov–Smirnov test. Continuous variables with normal distribution were expressed as mean ± standard deviation (SD), while non-normally distributed variables were presented as median (interquartile range). Categorical variables were expressed as percentages. Group comparisons were made using the chi-square test or Fisher’s exact test for categorical data, and the Student’s *t*-test or Mann–Whitney *U* test for continuous variables, as appropriate. The distribution of variables across different severity levels of early-onset PSD was visualized using box plots. Spearman rank correlation analysis was conducted to evaluate the relationships between variables and HAMD-17 scores across all patients. To assess for multicollinearity, we calculated the Variance Inflation Factor (VIF) for all independent variables. VIF values were all less than 5 (or 10), indicating no severe multicollinearity. If the VIF is indeed high, we will build two models for comparison: one containing NHHR but not its components (TC, HDL-C), and another containing TC and HDL-C but not NHHR. Binary logistic regression was performed to identify risk factors associated with early-onset PSD. Furthermore, sensitivity analyses were performed to test the robustness of our findings. The discriminative ability of NHHR in predicting early-onset PSD was evaluated using receiver operating characteristic (ROC) curve analysis. A two-tailed *p*-value of less than 0.05 was considered statistically significant. Statistical analyses were performed using SPSS software (version 25.0; IBM Corp.).

## Results

### Comparison of clinical and demographic characteristics between non-PSD and early-onset PSD patients

**Figure 2:**
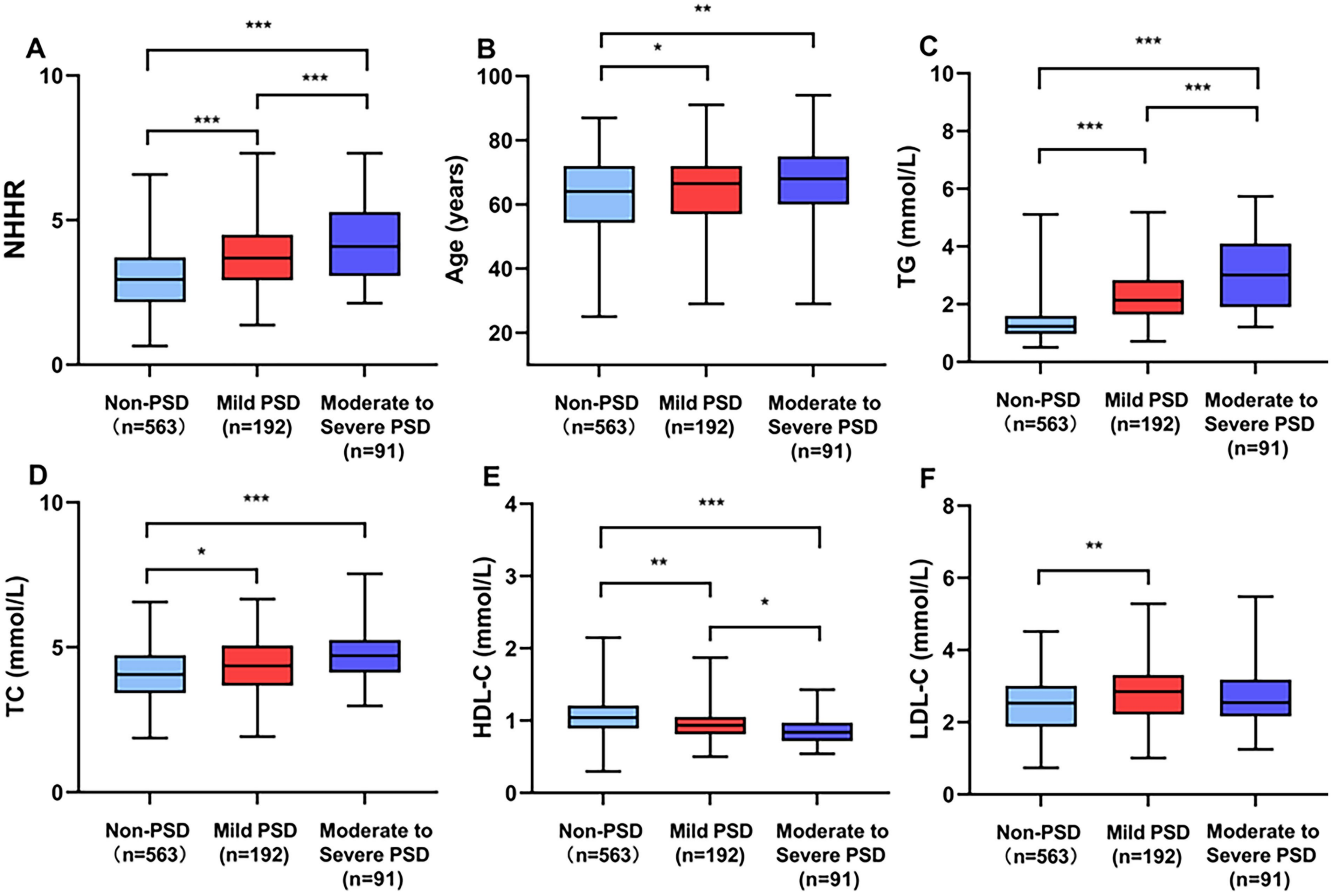
Comparing clinical biomarkers stratified by early-onset PSD severity. Box plots show the distribution of **(A)** NHHR, **(B)** age, **(C)** TG, **(D)** TC, **(E)** HDL-C, and **(F)** LDL-C. ***p < 0.001, **p < 0.01, *p < 0.05.

Table 1 presents a comprehensive overview of the clinical and demographic characteristics. This study observed 283 patients (33.45%) in the early-onset PSD group and 563 patients (66.55%) in the non-PSD group. The early-onset PSD group showed significantly lower in the percent of male patients (*p* < 0.001), BI scores (*p* < 0.001), and HDL-C (*p* < 0.001) than the non-PSD group, but significantly higher ages (*p* < 0.001), NIHSS scores (*p* = 0.001), mRS scores (*p* < 0.001), HAMD-17 scores (*p* < 0.001), TG (*p* < 0.001), TC (*p* < 0.001), LDL-C (*p* = 0.004), and NHHR (*p* < 0.001) than the non-PSD group. Additionally, Figure 2 illustrates the comparison of NHHR, age, TG, TC, HDL-C, and LDL-C among early-onset PSD categorized by varying severity levels.

**Table 1:** Clinical and demographic characteristics of the non-PSD and PSD groups.

Variable	Total (*n* = 846)	PSD (*n* = 283)	Non-PSD (*n* = 563)	*P*
Demographic characteristics				
Age, years	66.35 ± 11.64	67.52 ± 11.39	64.01 ± 11.81	<0.001
Male, *n* (%)	443 (66.42)	129 (57.33)	314 (71.04)	<0.001
BMI, kg/m^2^	23.50 ± 3.23	23.66 ± 3.35	23.34 ± 3.31	0.086
Vascular risk factors, *n* (%)				
Hypertension	679 (80.26)	235 (83.04)	444 (78.86)	0.150
Diabetes mellitus	215 (25.41)	74 (26.15)	141 (25.04)	0.728
Coronary artery disease	146 (17.26)	42 (14.84)	104 (18.47)	0.187
Current smoking	370 (43.74)	120 (42.40)	250 (44.40)	0.580
Current drinking	123 (26.36)	72 (25.44)	151 (26.82)	0.668
Stroke subtype, *n* (%)				0.983
LAA	223 (26.36)	75 (26.50)	148 (26.29)	
SAO	390 (46.10)	132 (46.64)	258 (45.83)	
CE	154 (18.20)	52 (18.37)	102 (18.12)	
SOE	34 (4.02)	10 (3.53)	24 (4.26)	
SUE	45 (5.32)	14 (4.95)	31 (5.51)	
Lesion location, *n* (%)				0.083
Anterior circulation	535 (63.24)	198 (69.96)	360 (63.94)	
Posterior circulation	264 (31.21)	67 (23.67)	174 (30.91)	
Both	48 (5.67)	18 (6.36)	29 (5.15)	
Medication use history, *n* (%)				
Previous antiplatelet	129 (15.25)	38 (13.43)	91 (16.16)	0.157
Previous statin	105 (12.41)	37 (13.07)	68 (12.08)	0.678
Previous antihypertension	629 (74.35)	217 (76.68)	412 (73.18)	0.272
Previous hypoglycemic agents	207 (24.47)	71 (25.09)	136 (24.16)	0.766
Neuropsychological evaluation				
NIHSS score, median (IQR)	2 (1–5)	3 (1–6)	2 (1–4)	0.001
mRS score, median (IQR)	1 (1–2)	2 (1–4)	1 (1–2)	<0.001
BI score, median (IQR)	85 (75–100)	80 (70–100)	94 (73–100)	<0.001
HAMD-17score, median (IQR)	5 (3–12)	16 (12–19)	4 (2–5)	<0.001
Laboratory data				
WBC (×10^9^/L)	6.89 ± 1.91	6.88 ± 1.89	6.91 ± 1.94	0.865
Cr (μmol/L)	75.34 ± 36.82	76.89 ± 38.87	74.67 ± 35.96	0.915
UA (μmol/L)	340.13 ± 94.52	342.31 ± 95.36	339 ± 94.48	0.741
TG (mmol/L)	1.46 (1.06–2.12)	2.15 (1.54–3.20)	1.24 (0.94–1.69)	<0.001
TC (mmol/L)	4.31 (3.59–4.99)	4.66 (3.98–5.29)	4.17 (3.48–4.85)	<0.001
HDL-C (mmol/L)	1.04 ± 0.27	0.97 ± 0.27	1.08 ± 0.26	<0.001
LDL-C (mmol/L)	2.62 ± 0.84	2.72 ± 0.87	2.52 ± 0.81	0.004
NHHR	3.21 (2.47–4.12)	3.86 (3.02–4.79)	2.96 (2.16–3.73)	<0.001

NHHR, non-high-density lipoprotein cholesterol to high-density lipoprotein cholesterol ratio; LDL-C, low-density lipoprotein cholesterol; HDL-C, high-density lipoprotein cholesterol; TC, total cholesterol; TG, triglyceride; UA, Uric acid; Cr, creatinine; WBC, white blood cell; HAMD-17, Hamilton depression scale 17 items; BI, Barthel Index; mRS, modified Rankin Scale; NIHSS, National Institutes of Health Stroke Scale; SUE, stroke of undetermined etiology; SOE, stroke of other determined etiology; CE, cardioembolism; SAO, small-artery occlusion; LAA, large-artery atherosclerosis.

### Associations between clinical variables and HAMD-17 scores

**Figure 3:**
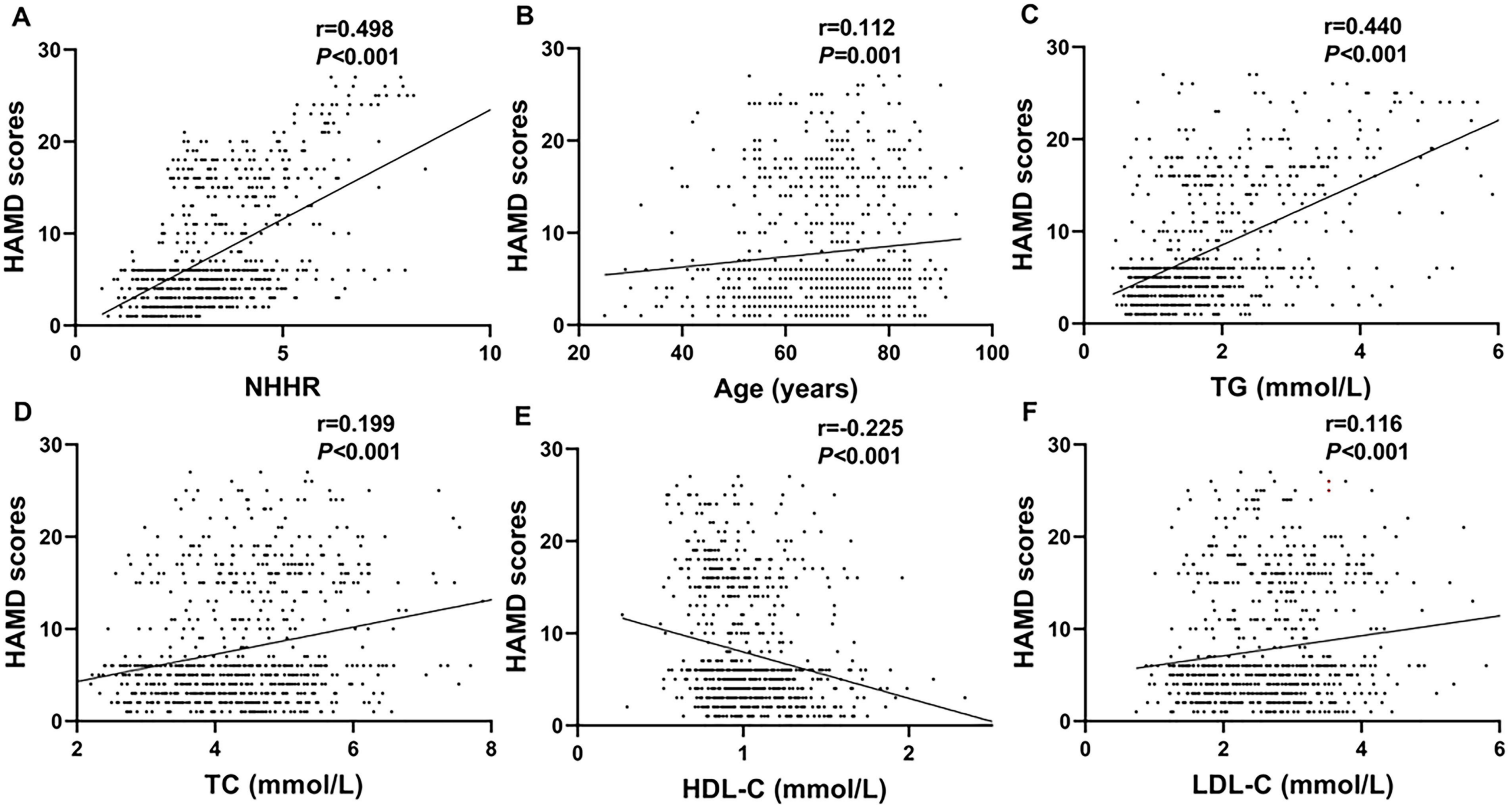
Scatter plots demonstrating correlations between clinical parameters and HAMD scores. Parameters positively correlated include NHHR (**A**; *r* = 0.498, *p* < 0.001), age (**B**; *r* = 0.112, *p* = 0.001), TG (**C**; *r* = 0.440, *p* < 0.001), TC (**D**; *r* = 0.199, *p* < 0.001), and LDL-C (**F**; *r* = 0.116, *p* < 0.001). The HDL-C (**E**; r = −0.225, *p* < 0.001) was negatively correlated.

A positive correlation was observed between HAMD-17 scores and NHHR (*r* = 0.498, *p* < 0.001), age (*r* = 0.112, *p* = 0.001), TG (*r* = 0.440, *p* < 0.001), TC (*r* = 0.199, *p* < 0.001), and LDL-C (*r* = 0.116, *p* < 0.001). Conversely, HAMD-17 scores showed a significant negative correlation with HDL-C (*r* = −0.225, *p* < 0.001) (Figure 3).

### Multivariate logistic regression analysis of determinants associated with early-onset PSD

A binary logistic regression model was employed to identify independent risk factors for early-onset PSD, using all variables that demonstrated statistical significance in Table 1. In addition, BMI was considered as a co-variate and has previously been associated with depression and should be included in multivariate analysis. To assess for multicollinearity, we calculated the VIF for all independent variables. However, there were collinearity between TC (VIF = 32), HDL-C (VIF = 25) and NHHR. We build two logistic regression analysis models for comparison: Table 2 containing NHHR but not its components (TC, HDL-C), and Table 3 containing TC and HDL-C but not NHHR. Multivariable logistic regression analysis revealed that a higher NHHR (OR, 1.796; 95% CI 1.452–1.996, *p* < 0.001) was significantly associated with an increased risk of early-onset PSD (Table 2). In this study, TC and HDL-C were the constituent variables of NHHR, but were not independent risk factors or protective factors for early-onset PSD (Table 3). In addition, after stratifying NHHR into tertiles and adjusting for confounders, patients exhibiting elevated NHHR levels (3rd quartile vs. 1st quartile; OR, 1.743; 95% CI, 1.411–2.042, *p* = 0.005) demonstrated a heightened risk of early-onset PSD (Table 4).

**Table 2:** Logistic regression analysis for early-onset PSD, containing NHHR but not its components (TC, HDL-C).

Variable	OR (95% CI)	*P*	Adjusted OR (95% CI)	*P*
Age	1.276 (1.055–1.421)	0.002	1.141 (1.002–1.361)	0.360
Male	0.832 (0.636–0.993)	0.005	0.742 (0.471–0.904)	0.101
BMI	1.132 (1.043–1.327)	0.075	1.154 (1.056–1.432)	0.231
NIHSS score	1.912 (1.735–2.104)	0.018	1.743 (1.509–1.913)	0.470
mRS score	1.602 (1.203–1.832)	0.007	1.533 (1.351–1.932)	0.091
BI score	1.442 (1.037–1.628)	0.002	1.292 (1.150–1.491)	0.431
LDL-C	1.423 (1.276–1.703)	0.020	1.220 (1.052–1.447)	0.123
TG	1.943 (1.072–2.213)	<0.001	1.752 (1.491–1.975)	0.055
NHHR	2.241 (1.854–2.542)	<0.001	1.796 (1.452–1.996)	<0.001

**Table 3:** Logistic regression analysis for early-onset PSD, containing TC and HDL-C but not NHHR.

Variable	OR (95% CI)	*P*	Adjusted OR (95% CI)	*P*
Age	1.276 (1.055–1.421)	0.002	1.139 (1.001–1.346)	0.237
Male	0.832 (0.636–0.993)	0.005	0.751 (0.459–0.936)	0.114
BMI	1.132 (1.043–1.327)	0.075	1.231 (1.209–1.405)	0.113
NIHSS score	1.912 (1.735–2.104)	0.018	1.751 (1.497–1.974)	0.371
mRS score	1.602 (1.203–1.832)	0.007	1.505 (1.307–1.742)	0.275
BI score	1.442 (1.037–1.628)	0.002	1.363 (1.098–1.512)	0.240
LDL-C	1.423 (1.276–1.703)	0.020	1.367 (1.134–1.589)	0.531
TG	1.943 (1.072–2.213)	<0.001	1.778 (1.229–1.945)	0.079
TC	1.484 (1.270–1.739)	0.015	1.361 (1.298–1.640)	0.195
HDL-C	0.824 (0.659–0.959)	<0.001	0.765 (0.591–0.901)	0.062

**Table 4:** Association of NHHR with early-onset PSD.

Variable	OR (95% CI)	*P*	Adjusted OR (95% CI)^a^	*P*
NHHR ternary classification				
T1	Reference		Reference	
T2	1.873 (1.321–2.114)	<0.001	1.615 (1.317–1.935)	0.001
T3	2.001 (1.714–2.213)	<0.001	1.743 (1.411–2.042)	0.005

NHHR: T1 ≤ 2.73; 2.73 < T2 ≤ 3.79; T3 > 3.79. ^a^Model: adjusted for age, male, initial NIHSS score, mRS score, BI score, TG, and LDL-C.

### Subgroup analyses and interaction test

**Figure 4:**
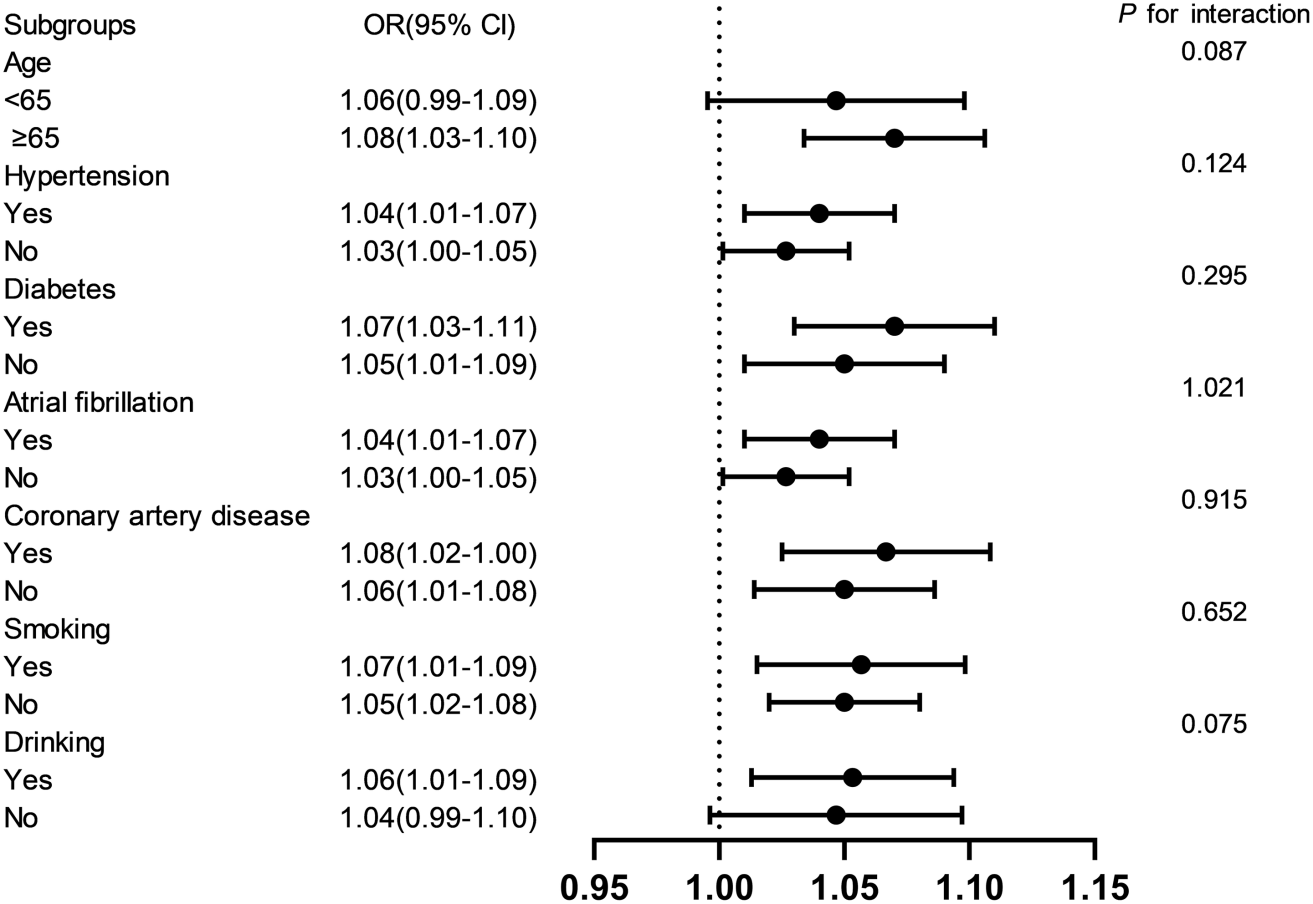
Subgroup analysis for the association between the NHHR with early-onset PSD.

Stratified analyses were performed to evaluate the consistency of the association between NHHR and early-onset PSD across key demographic and clinical subgroups (Figure 4). Results demonstrated that the positive association remained robust across subgroups stratified by age (<65, ≥65 years), diabetes mellitus, hypertension, coronary artery disease, atrial fibrillation, alcohol consumption, and smoking status. Interaction tests revealed no statistically significant effect modification by any of these covariates (*p* > 0.05), indicating that the relationship between NHHR and early-onset PSD was not significantly influenced by these factors.

### The discriminative ability of NHHR for early-onset PSD was evaluated using ROC curve analysis

**Figure 5:**
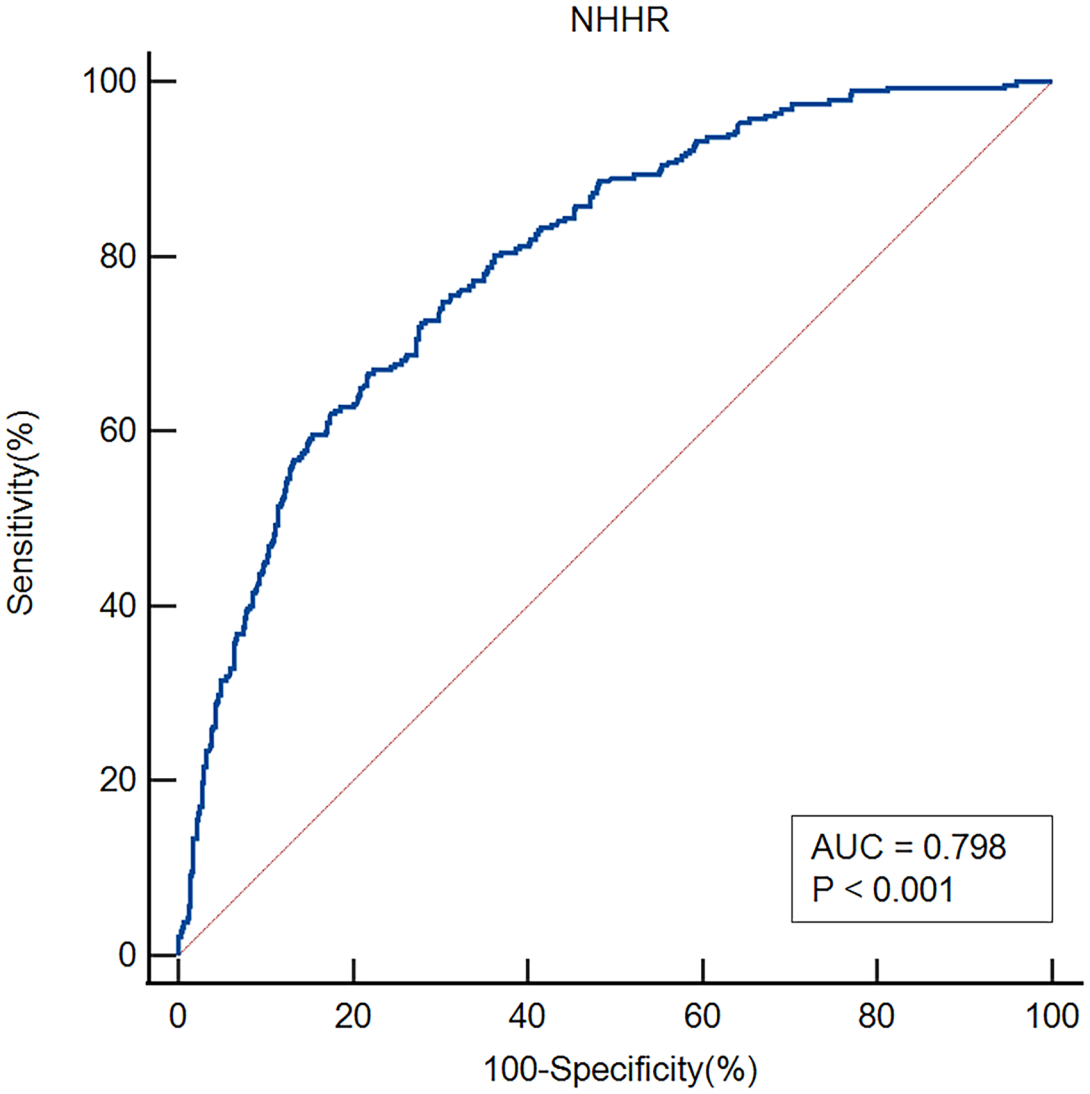
ROC curve of NHHR for predicting early-onset post-stroke depression (AUC = 0.798).

The ROC analysis was used to evaluate the ability of NHHR to discriminate patients with early-onset PSD (Figure 5). The area under the curve (AUC) for NHHR was 0.798 (95% CI: 0.769–0.824; *p* < 0.001). At the optimal cut-off value of 3.56, sensitivity was 66.67% and specificity was 78.15%.

## Discussion

Researchers have extensively studied the associations between serum lipids and stroke, along with post-stroke depression complications. PSD is the most common psychiatric comorbidity after stroke; given the significant impact of PSD on individuals and society, identifying and managing modifiable risk factors is crucial (29). This study examines the correlation between NHHR and early-onset PSD. This study produced several new findings. Patients in the early-onset PSD group initially demonstrated significantly higher NHHR, NIHSS, and mRS scores, along with lower BI scores, compared to those in the non-PSD group. HAMD-17 scores were positively correlated with NHHR levels. Logistic regression identified NHHR as an independent risk factor for early-onset PSD. Furthermore, subgroup analyses revealed no significant interactions (all *p* > 0.05), supporting the consistency of the association between NHHR and early-onset PSD across different patient subgroups and underscoring the robustness and broad applicability of our findings. Finally, ROC analysis indicated that NHHR has clinically meaningful discriminatory power for early-onset PSD. The results demonstrate a correlation between elevated NHHR and early-onset PSD.

There is evidence linking the risk of PSD to the dysregulation of lipid profiles. Higher LDL-C/HDL-C ratios and lower HDL-C levels are associated with a higher risk of PSD (16). Elevated MHR was strongly linked to an increased risk of PSD, according to another cross-sectional investigation (17), which indirectly confirmed that lower levels of HDL-C were associated with increased risk of PSD. However, a retrospective study with 8,207 participants indicated a significant association between higher levels of HDL-C and an increased risk of depressive symptoms (30). Finding new biomarkers to evaluate the risk of early-onset PSD is crucial to improving preventative and treatment approaches because lipids have not been thoroughly taken into account in previous research. The NHHR is a developing comprehensive metric of atherosclerotic lipids (31). This ratio provides a more comprehensive assessment of atherosclerotic potential than traditional lipid parameters (32). Prior research has demonstrated that when assessing the risk of non-alcoholic fatty liver disease (33) and chronic kidney disease (34), the NHHR performs better in terms of predictive and diagnostic capabilities than conventional blood lipid levels. Numerous recent studies have highlighted NHHR’s predictive worth and its strong prognostic value in people with cardiovascular disease (1, 35–37). A prior study indicated that NHHR is associated with an increased prevalence of stroke and may become a new predictor of stroke (38). Moreover, there have been several previous studies that have shown the relationship between NHHR and depression. Elevated NHHR levels were significantly correlated with an increased likelihood of suicidal ideation (22). In American adults, NHHR was substantially linked to an increased risk of depression (20). Recently, a cross-sectional study indicated that NHHR was significantly correlated with an increased risk of PSD among U. S. adults (25). Further large-scale prospective studies are needed to confirm the causal relationship between NHHR and PSD. To date, few studies have specifically analyzed the role of NHHR in predicting the risk of early-onset PSD. In our study, 283 patients (33.45%) experienced early-onset PSD, and the proportion was consistent with the results of previous studies (10, 39). The NHHR had a positive relationship with the severity of early-onset PSD. There were collinearity between TC, HDL-C and NHHR. We build two logistic regression analysis models for comparison: one containing NHHR but not its components (TC, HDL-C), and another containing TC and HDL-C but not NHHR. In this study, TC and HDL-C were the constituent variables of NHHR, but were not independent risk factors or protective factors for early-onset PSD. After adjusting for potential confounders, binary logistic regression confirmed that NHHR served as an independent predictor of early-onset PSD. When analyzed as a tertile-based categorical variable, elevated NHHR levels remained significantly associated with increased risk of early-onset PSD. Moreover, NHHR demonstrated good discriminative ability for early-onset PSD, with an AUC of 0.798. This study provides new insights into the role of NHHR in PSD risk and supports its potential as a novel indicator for early-onset PSD risk assessment.

The underlying mechanism by which NHHR increases the risk of early-onset PSD remains incompletely understood. However, several potential pathophysiological pathways may help explain this association. Firstly, inflammatory reactions may be triggered or made worse by lipid abnormalities, specifically low levels of HDL-C and high levels of LDL-C. Numerous pro-inflammatory cytokines can be released by inflammatory reactions following an acute stroke, according to studies, which may raise the likelihood of depressive symptoms (40). Furthermore, oxidative stress is thought to be a major contributor to PSD pathogenesis. Lipid and protein peroxidation brought on by oxidative stress can impact neurotransmitter synthesis and metabolism, resulting in pathogenic alterations in the nervous system (41, 42). Low HDL-C levels may impair antioxidant and anti-inflammatory properties, leaving the brain more vulnerable to these damaging processes (43, 44). Therefore, through pathways including oxidative stress and inflammation, alterations in the lipid profile may raise the risk of PSD. In addition, studies on lipid-related regulation of neurovascular integrity, microglial activation, and neuroendocrine dysregulation have been reported. The vessel-adjacent microglia were specifically activated by the leakage of plasma low-density lipoprotein (LDL), which led to blood–brain barrier (BBB) breakdown and ischemic demyelination (45). A recent study showed that palmitoylation of microglial protein kinase Cδ (PKCδ) in the hypothalamus plays a role in modulating peripheral lipid metabolism through hypothalamus-liver communication (46). Of particular interest, recent work has shown that specific lipid–glucose interactions, modulated by genetic background, can shape vascular injury distribution and systemic metabolic profiles (47). These data support the concept that composite lipid markers like NHHR may act as integrators of metabolic and vascular risk pathways. Our research in order to strengthen and optimize the understanding of NHHR and early-onset PSD between risk provides a new perspective and strong evidence. In addition to conventional lipid-lowering therapies, novel agents such as PCSK9 inhibitors and inclisiran have been shown to exert anti-inflammatory and endothelial-protective effects (48), which could potentially mitigate PSD risk in dyslipidemic stroke survivors. Similarly, lifestyle interventions and dietary patterns that improve NHHR can also reduce the risk of early-onset PSD.

Furthermore, patients in the early-onset PSD group exhibited higher NIHSS and mRS scores, along with lower BI scores, indicating more severe stroke-related impairment and functional disability. Unfavorable physical conditions may worsen psychological problems, including depression (49). According to this study, early-onset PSD was more common in female stroke patients than in male ones. Some potential solutions include social factors, such as exposure to gender-specific stressors; psychological factors, such as gender-specific symptom profiles; and physiological factors, such as genetic variances and sex hormones (50). Multiple studies have identified a strong association between atherogenic factors and depression. A previous study has shown that the level of TG was one of the most important features in predicting depression (51). However, another study reported conflicting results, TG was negatively correlated with depressive symptom severity (52). At present, the relationship between TC and depression is not clear. Low levels of TC were not associated with an increased risk of depression (53), while other studies reported an inverse relationship between depression and levels of TC (52, 54–56). In addition, a recent study showed that high HDL-C levels were negatively associated with depression (57). In our study, TG, TC or HDL-C were not independent risk factors or protective factors for early-onset PSD. We propose that discrepancies across studies may be attributed to differences in the ethnic composition of study populations, limited sample sizes, variations in medication use, and heterogeneity in disease severity.

The following are the limitations of this study: (1) Several risk factors that could influence depressive episodes, such as mounting life stress, educational background, and social support, were not included in our study; (2) patients who experienced severe aphasia, coma, or dementia while in the hospital were excluded; (3) this study was a single-center study and only included Chinese patients, suggesting the presence of potential inherent biases. Consequently, future research should multicenter validation across different geographic and ethnic groups would reinforce external validity; (4) TC and HDL-C are the component variables of NHHR and may themselves be independent risk factors or protective factors; so, the study findings still need to be further confirmed by multi-center and large-sample clinical studies; (5) future research needs to integrating circulating biomarkers such as high-sensitivity C-reactive protein, IL-6, or neurotrophic factors (e.g., brain-derived neurotrophic factor) could refine risk stratification and help elucidate causal pathways; and (6) conclusions from short-term observational studies may not be comprehensive enough.

## Conclusion

According to our research, NHHR can be utilized as a predictive indicator and may be an independent risk factor for early-onset PSD. There are important therapeutic ramifications for early-onset PSD screening, treatment plan formulation, etiology research, and prognosis evaluation if an association between NHHR and early-onset PSD is discovered. Incorporating NHHR into assessment and treatment strategies is expected to improve overall clinical outcomes for patients. However, to precisely understand the role of NHHR in the pathophysiology of early-onset PSD, more research is necessary.

## Data Availability

The raw data supporting the conclusions of this article will be made available by the authors, without undue reservation.
